# Unraveling the molecular mechanisms of lymph node metastasis in ovarian cancer: focus on MEOX1

**DOI:** 10.1186/s13048-024-01384-6

**Published:** 2024-03-14

**Authors:** Jiajia Li, Yihua Sun, Xiuling Zhi, Yating Sun, Zulimire Abudousalamu, Qianhan Lin, Bin Li, Liangqing Yao, Mo Chen

**Affiliations:** 1 Department of Gynecology Oncology, Obstetrics & Gynecology Hospital, Fudan University, Shanghai, 200011 China; 2 Department of Pathology, Obstetrics and Gynecology Hospital of Fudan University, Shanghai, 200011 China; 3 Department of Physiology and Pathophysiology, School of Basic Medical Sciences, Fudan University, Shanghai, 200032 China

**Keywords:** Ovarian cancer, Lymph node metastasis, MEOX1, Lymphangiogenesis, Bioinformatics

## Abstract

**Background:**

Lymph node metastasis (LNM) is a major factor contributing to the high mortality rate of ovarian cancer, making the treatment of this disease challenging. However, the molecular mechanism underlying LNM in ovarian cancer is still not well understood, posing a significant obstacle to overcome.

**Results:**

Through data mining from The Cancer Genome Atlas (TCGA) and Gene Expression Omnibus (GEO) databases, we have identified MEOX1 as a specific gene associated with LNM in ovarian cancer. The expression of MEOX1 was found to be relatively high in serous ovarian adenocarcinoma, and its higher expression were associated with increased tumor grade and poorer clinical prognosis for ovarian cancer patients. Bioinformatics analysis revealed that MEOX1 exhibited the highest mRNA levels among all cancer types in ovarian cancer tissues and cell lines. Furthermore, gene set enrichment analysis (GSEA) and pathway analysis demonstrated that MEOX1 was involved in various LNM-related biological activities, such as lymphangiogenesis, lymphatic vessel formation during metastasis, epithelial-mesenchymal transition (EMT), G2/M checkpoint, degradation of extracellular matrix, and collagen formation. Additionally, the expression of MEOX1 was positively correlated with the expression of numerous prolymphangiogenic factors in ovarian cancer. To validate our findings, we conducted experiments using clinical tissue specimens and cell lines, which confirmed that MEOX1 was highly expressed in high-grade serous ovarian cancer (HGSOC) tissues and various ovarian cancer cell lines (A2780, SKOV3, HO8910, and OVCAR5) compared to normal ovarian tissues and normal ovarian epithelial cell line IOSE-80, respectively. Notably, we observed a higher protein level of MEOX1 in tumor tissues of LNM-positive HGSOC compared to LNM-negative HGSOC. Moreover, our fundamental experiments demonstrated that suppression of MEOX1 led to inhibitory effects on ovarian cancer cell proliferation and EMT, while overexpression of MEOX1 enhanced the proliferation and EMT capacities of ovarian cancer cells.

**Conclusions:**

The results of our study indicate that MEOX1 plays a role in the lymph node metastasis of ovarian cancer by regulating multiple biological activities, including the proliferation and EMT of ovarian cancer, lymphangiogenesis, and ECM remodeling. Our findings suggest that MEOX1 could serve as a potential biomarker for the diagnosis and treatment of ovarian cancer with LNM.

**Supplementary Information:**

The online version contains supplementary material available at 10.1186/s13048-024-01384-6.

## Introduction

As one of the most prevalent gynecologic malignancies, ovarian cancer is known for its worst clinical prognosis and highest mortality rate [1]. Lymph node metastasis (LNM) is a critical factor contributing to the high mortality of ovarian cancer and directly affects clinical staging and patient prognostic outcomes [2–4]. The rate of LNM positivity in high-grade serous ovarian cancer (HGSOC) increases from 25% in stage I-II patients to 75% in stage III-IV patients [5, 6]. Additionally, the detection of positive lymph nodes can lead to the classification of early-stage ovarian cancer patients as stage III-IV based on postoperative pathological examination [3]. Notably, ovarian cancer patients with LNM suffer a higher risk of recurrence and death compared to patients without LNM [4]. Despite significant advancements in ovarian cancer surgery and chemotherapy in the past few decades, LNM still poses a challenge to treatment [7–9]. In recent years, tumor-targeted therapy has gained increasing attention for its advantages, such as low side effects, high patient tolerance, and more precise anti-tumor effects [10]. Targeted inhibition of LNM could be a potential approach for treating ovarian cancer, but the molecular mechanism underlying LNM in ovarian cancer remains elusive [11, 12]. Therefore, there is an urgent need to elucidate the molecular mechanisms of ovarian cancer LNM and identify more specific and reliable LNM-related biomarkers through broader genomic and biological research on ovarian cancer.

With the rapid advancement of genome sequencing technology, there is an increasing abundance of publicly available cancer genome database resources. By utilizing multi-platform genomic high-throughput sequencing data and corresponding clinical information, we can uncover the pathological mechanisms of tumor occurrence and development and identify new molecular targets for accurate diagnosis and treatment of malignancies [13, 14]. In the present study, we screened out an LNM-related molecule in ovarian cancer, mesenchyme homeobox 1 (MEOX1), through data mining from the Cancer Genome Atlas TCGA (TCGA) and Gene Expression Omnibus (GEO) databases. MEOX1, a member of the homeobox gene family, was initially discovered in the 17q21 region of the breast cancer susceptibility protein 1 (BRCA1) [15]. As an essential transcription factor involved in embryonic development, MEOX1 plays a role in the formation of somites [16], promotes injury-induced vascular re-modeling [14], and accelerates myocardial hypertrophy [17]. While most studies have focused on embryonic development, recent research has shown that MEOX1 is abnormally expressed in various tumor tissues and contributes to various phenotypes associated with tumor progressions, such as tumor proliferation, metastasis, and chemoresistance [18–22]. However, the specific role and mechanism of MEOX1 in ovarian cancer, particularly in LNM, remain obscure and require further investigation.

In this study, we first conducted an examination of the expression of MEOX1 in ovarian cancer and its correlation with LNM using bioinformatics methods, cell lines, and clinical specimens. Subsequently, we performed further investigations to explore the impact of MEOX1 on the biological activities of ovarian cancer cells both in vitro and in vivo.

## Results

### Identification of lymph node metastasis-related genes in ovarian cancer

**Fig. 1:**
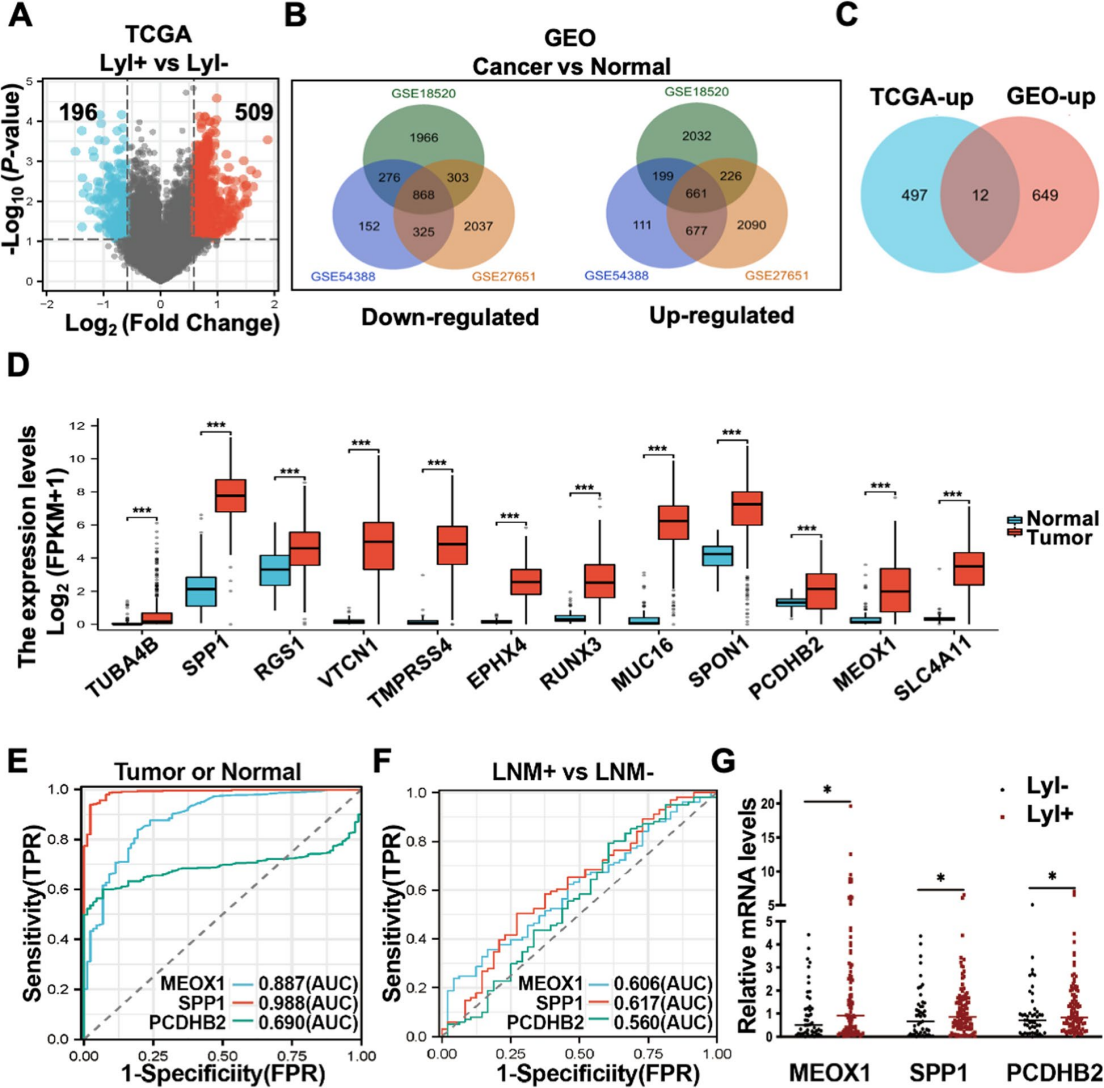
Identification of LNM-Related Genes in Ovarian Cancer. **A** The volcano map of 509 upregulated LyI-related DEGs (indicated as red dots) and 196 downregulated LyI-related DEGs (indicated as blue dots) (LyI + vs. LyI-; |Fold Change |> 1.5, *P*-value < 0.05) of TCGA ovarian cancer. **B** The overlap of DEGs between ovarian cancer tissues and normal ovarian tissues of GSE18520, GSE54388, and GSE27651 from the GEO database. **C** The overlap of 509 LyI-related up-regulated DEGs of the TCGA database and 661 cancer-related up-regulated DEGs of the GEO database resulted in 12 genes. **D** The relative mRNA levels of the overlapping 12 genes in ovarian cancer tissues (*n* = 427, the TCGA database) and normal ovarian tissues (*n* = 88, the GTEx database); ***P < 0.001. E–F. ROC curve analysis of MEOX1, SPP1, and PCDHB2 in predicting the sample status (normal or tumor) (**E**) and lymph node metastatic status (LNM + or LNM-) (F) of TCGA ovarian cancer; the value of AUC indicates the diagnostic effect. G. The relative mRNA expression of MEOX1, SPP1, and PCDHB2 in LyI + and LyI- TCGA ovarian cancer; * *P* < 0.05

Recent studies have shown that the genetic abnormalities found in lymph node metastases are mostly already present in the primary tumor tissues, with only a few occurring exclusively in metastatic lesions [23, 24]. In this study, we focused on identifying LNM-related genes in ovarian cancer by analyzing primary tumor lesions from public databases. We obtained 99 lymphatic invasion (LyI) positive (LyI+) and 47 LyI negative (LyI-) ovarian cancer expression profiles from the TCGA database, along with corresponding clinical data. The baseline characteristics of the patients, including Federation of International of Gynecologists and Obstetricians (FIGO) staging, vital status, histopathology, patient's race, and age, were well balanced between the LyI+ and LyI- groups (Table 1). We then compared the gene expression profiles between the LyI+ and LyI- groups and identified 196 downregulated differentially expressed genes (DEGs) (shown as blue dots) and 509 upregulated DEGs (shown as red dots) (Figure. 1A, Supplementary Table S1).

**Table 1:** The baseline characteristics between LyI- and LyI + TCGA ovarian cancer patients

Variable	LyI-^a^	LyI + ^b^	*P*
**n**	47	99	
**Age, mean ± SD**	58.19 ± 12.01	58.13 ± 10.81	0.976
**Race, n (%)**			0.825
White	39 (83%)	86 (86.9%)	
Asian	3 (6.4%)	3 (3%)	
Black or African American	3 (6.4%)	5 (5.1%)	
Native Hawaiian or other pacific islander	0 (0%)	1 (1%)	
Not reported	2 (4.3%)	4 (4%)	
**FIGO**^**c**^** stage, n (%)**			0.843
I-II	6 (12.8%)	10 (10.1%)	
III-IV	41 (87.2%)	89 (89.9%)	
**Vital status, n (%)**			0.790
Alive	27 (57.4%)	53 (53.5%)	
Dead	20 (42.6%)	46 (46.5%)	
**Histological subtype, n (%)**			0.322
Papillary serous cystadenocarcinoma	1 (2.1%)	0 (0%)	
Serous cystadenocarcinoma	46 (97.9%)	99 (100%)	

^a^lymphatic invasion negative

^b^lymphatic invasion positive

^c^Federation of International of Gynecologists and Obstetricians

To identify genes closely associated with the development and progression of ovarian cancer, we compared gene expression profiles between ovarian cancer tissues and normal ovarian tissues to screen for cancer-related DEGs by using three gene expression profiles of ovarian cancer (GSE18520, GSE54388, and GSE27651) from the GEO database (Supplementary Figure S1, Supplementary Table S2). We obtained 661 upregulated and 868 downregulated cancer-related DEGs in all three profiles (Figure. 1B). Subsequently, the intersection of 509 upregulated LyI-related DEGs with 661 upregulated cancer-related DEGs yielded 12 candidate LNM-related genes for ovarian cancer (Figure. 1C). To validate our findings, we further corroborated the aberrant upregulation of each of the 12 candidate genes in ovarian cancer tissues using the TCGA and GTEx databases (Figure. 1D).

Only high expression of MEOX1(205619_s_at), secreted phosphoprotein 1(SPP1, 209875_s_at), and procalcitonin β2 (PCDHB2, 231725_at) were found to be significantly associated with adverse overall survival (OS) and progression-free survival (PFS) of ovarian cancer patients, as determined by the prognostic analysis via the Kaplan-Meier plotter (Table 2). To further evaluate the discriminatory capacity of these three genes in distinguishing between tumor tissues of ovarian cancer and normal ovarian tissues, as well as differentiating between LNM+ ovarian cancer and LNM- ovarian cancer, we conducted receiver operating characteristic (ROC) analysis by calculating the area under the curve (AUC). The ROC analysis revealed that both MEOX1 (AUC = 0.887, Confidence interval (CI) = 0.849 - 0.926) and SPP1 (AUC = 0.988, CI = 0.979 - 0.997) expression could serve as single significant parameters for discriminating between normal and tumor tissues of ovarian cancer (Figure. 1E). However, MEOX1 (AUC = 0.606, CI = 0.512 - 0.701) and SPP1 (AUC = 0.617, CI = 0.519 - 0.716) exhibited limited discriminatory capacity in differentiating between LNM+ and LNM- ovarian cancer (Figure. 1F). PCDHB2 showed some predictive ability in determining the status of samples (normal or tumor, AUC = 0.690, CI = 0.648 - 0.732) (Figure. 1E) but had a lower predictive ability in predicting lymph node metastatic status (LNM+ or LNM-, AUC = 0.560, CI = 0.455 - 0.665) in ovarian cancer (Figure. 1F). Among the three genes, MEOX1 exhibited the highest differential expression between the LyI+ group and the LyI- group (Figure. 1G). Thus, we ultimately selected MEOX1 as the molecule for further research. In addition, we compared the predictive value for LNM in ovarian cancer of MEOX1 with commonly used ovarian cancer recurrence and invasive biomarkers in clinical practice, including CA125 (MUC16), CA153 (MUC1), CEA (CEACAM5), and HE4 (WFDC2) using the TCGA database. We found that MEOX1's predictive ability (AUC = 0.606) was second only to CA125 (AUC = 0.640) (Supplementary Figure S2A), while MEOX1 showed the greatest difference in expression between LNM+ and LNM- ovarian cancer tissues (Supplementary Figure S2B). Therefore, it can be concluded that MEOX1 may have a high clinical significance in diagnosing and predicting LNM in ovarian cancer.

**Table 2:** Prognostic analysis of 12 candidate LNM-related genes in ovarian cancer

Gene	OS		PFS	
	**HR**^**a**^** ( 95%CI**^**b**^**)**	***P***	**HR**^**a**^** ( 95%CI**^**b**^**)**	***P***
TUBA4B	0.84 (0.74, 0.96)	0.010	0.88 (0.78, 1.01)	0.062
SPP1	1.31 (1.16, 1.50)	< 0.001	1.38 (1.21, 1.57)	< 0.001
RGS1	0.93 (0.82, 1.06)	0.010	1.25 (1.09, 1.42)	< 0.001
VTCN1	1.12 (0.97, 1.30)	0.130	1.15 (0.99, 1.33)	0.066
TMPRSS4	0.83 (0.73, 0.95)	0.006	0.83 (0.73, 0.95)	0.008
EPHX4	0.85 (0.69, 1.05)	0.130	1.31 (1.05, 1.65)	0.019
RUNX3	0.83 (0.72, 0.97)	0.019	0.94 (0.82, 1.09)	0.430
MUC16	0.83 (0.73, 0.95)	0.005	1.21 (1.05, 1.41)	0.010
SPON1	0.83 (0.73, 0.94)	0.005	0.91 (0.79, 1.05)	0.210
PCDHB2	1.31 (1.07, 1.62)	0.010	1.49 (1.21, 1.84)	< 0.001
MEOX1	1.16 (1.02, 1.32)	0.021	1.21 (1.06, 1.38)	0.005
SLC4A11	0.79 (0.64, 0.97)	0.024	1.36 (1.11, 1.68)	0.003

^a^Hazard Ratio; > 1 represents a poor prognostic factor, and < 1 represents a good prognostic factor

^b^Condifence Interval

### Pancancer analysis revealed that MEOX1 had the highest expression in ovarian cancer

**Fig. 2:**
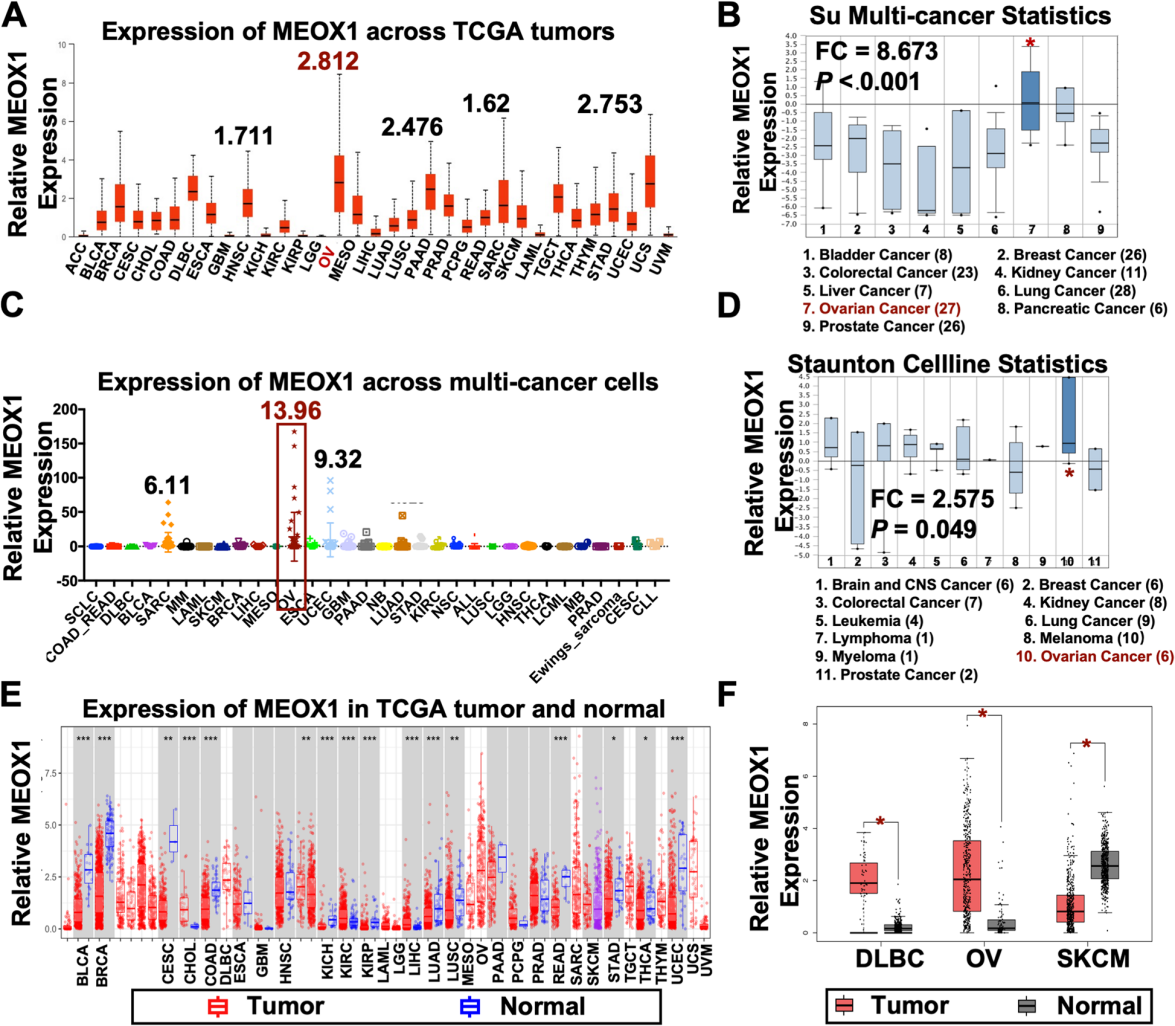
Pan-cancer Analysis of MEOX1 Expression in various cancer types. **A** The mRNA expression of MEOX1 in a wide array of TCGA cancer types from the UALCAN portal. **B** The relative mRNA expression level of MEOX1 in various cancer types from the Oncomine; *P < 0.05 vs. Kidney Cancer. **C** The relative mRNA level of MEOX1 across multi-cancer cells derived from the CCLE database. **D** The relative mRNA expression level of MEOX1 in multi-cancer cells from the Oncomine; *P < 0.05 vs. Melanoma. **E** The relative mRNA level of MEOX1 in pan-cancer tumor tissues (red columns) and the corresponding TCGA normal controls (blue columns) from the TIMER.2.0 database; *P < 0.05, **P < 0.01, ***P < 0.001 vs. normal controls. **F** The mRNA expression of MEOX1 in tumor tissues (red columns) and matched GTEx normal tissues (grey columns) of DLBC, OV, and SKCM as analyzed by the GEPIA2.0; *P < 0.05

In our study, we investigated the expression of MEOX1 in various cancer types using different databases. Firstly, we utilized the University of ALabama at Birmingham Cancer Data Analysis Portal (UALCAN) to analyze the median mRNA expression of MEOX1 in tumor tissues. Interestingly, we noticed that ovarian cancer exhibited the highest median mRNA expression of MEOX1 compared to other cancer types (Figure. 2A). These findings were consistent with the Su Multi-cancer statistics (Reporter: 36010_at) obtained from the Oncomine database, which also demonstrated that the median mRNA expression of MEOX1 in tumor tissues of ovarian cancer ranked first among all cancer types (Figure. 2B). Moreover, we explored the Cancer Cell Line Encyclopedia (CCLE) database and the Oncomine database (Staunton Cellline Statistics, reporter: U10492_at) to assess the mRNA levels of MEOX1 in cancer cell lines. Remarkably, ovarian cancer cell lines showed the highest average mRNA level of MEOX1 compared to other cancer types (Figure. 2C-D).

Next, we investigated the expression of MEOX1 in tumor tissues and matched normal tissues of various TCGA cancer types using two databases: Tumor Immune Estimation Resource Version 2 (TIMER2.0) and Gene Expression Profiling Interactive Analysis version 2 (GEPIA2.0). Our analysis revealed that MEOX1 appeared to be downregulated in various tumor tissues including bladder urothelial carcinoma (BLCA), breast invasive carcinoma (BRCA), cervical squamous cell carcinoma and endocervical adenocarcinoma (CESC), colon adenocarcinoma (COAD), kidney chromophobe (KICH), kidney renal papillary cell carcinoma (KIRP), lung adenocarcinoma (LUAD), lung squamous cell carcinoma (LUSC), rectum adenocarcinoma (READ), stomach adenocarcinoma (STAD), thyroid carcinoma (THCA), and uterine corpus endometrial carcinoma (UCEC) compared to the corresponding normal tissues, except for cholangiocarcinoma (CHOL), kidney renal clear cell carcinoma (KIRC), and liver hepatocellular carcinoma (LIHC) (Figure. 2E). However, due to the unavailability of matched normal controls in the TIMER2.0 database for certain cancer types such as ovarian cancer (OV), diffuse large B-cell lymphoma (DLBC), acute myeloid leukemia (LAML), brain lower-grade glioma (LGG), sarcoma (SARC), testicular germ cell tumors (TGCT), thymoma (THYM), uterine carcinosarcoma (UCS), and skin cutaneous melanoma (SKCM), we turned to the GEPIA2.0 database with matching TCGA normal and GTEx data as controls. Our findings demonstrated that the expression of MEOX1 in tumor tissues and normal tissues was statistically different only in DLBC, OV, and SKCM (Supplementary Figure S3, Figure. 2F). Specifically, MEOX1 was found to be overexpressed in tumor tissues of DLBC and OV, while its expression was significantly decreased in tumor tissues of SKCM (Figure. 2F).

In addition, a lower level of MEOX1 was found to be associated with shorter OS in LUSC, LUAD, BRCA, and CESC (Supplementary Figure S4A), and shorter PFS in LUSC and CESC (Supplementary Figure S4B). We reasoned that MEOX1 may act as an antioncogene in LUSC, LUAD, BRCA, and CESC due to its downregulation in tumors and its association with poor survival. Conversely, the overexpression of MEOX1 in ovarian cancer and its correlation with an unfavorable prognosis suggested that MEOX1 might function as an oncogene in ovarian cancer. Based on these findings, we propose that MEOX1 may play a role in the occurrence and progression of ovarian cancer.

### Bioinformatics analysis revealed that MEOX1 was highly expressed in high-grade serous ovarian cancer

**Fig. 3:**
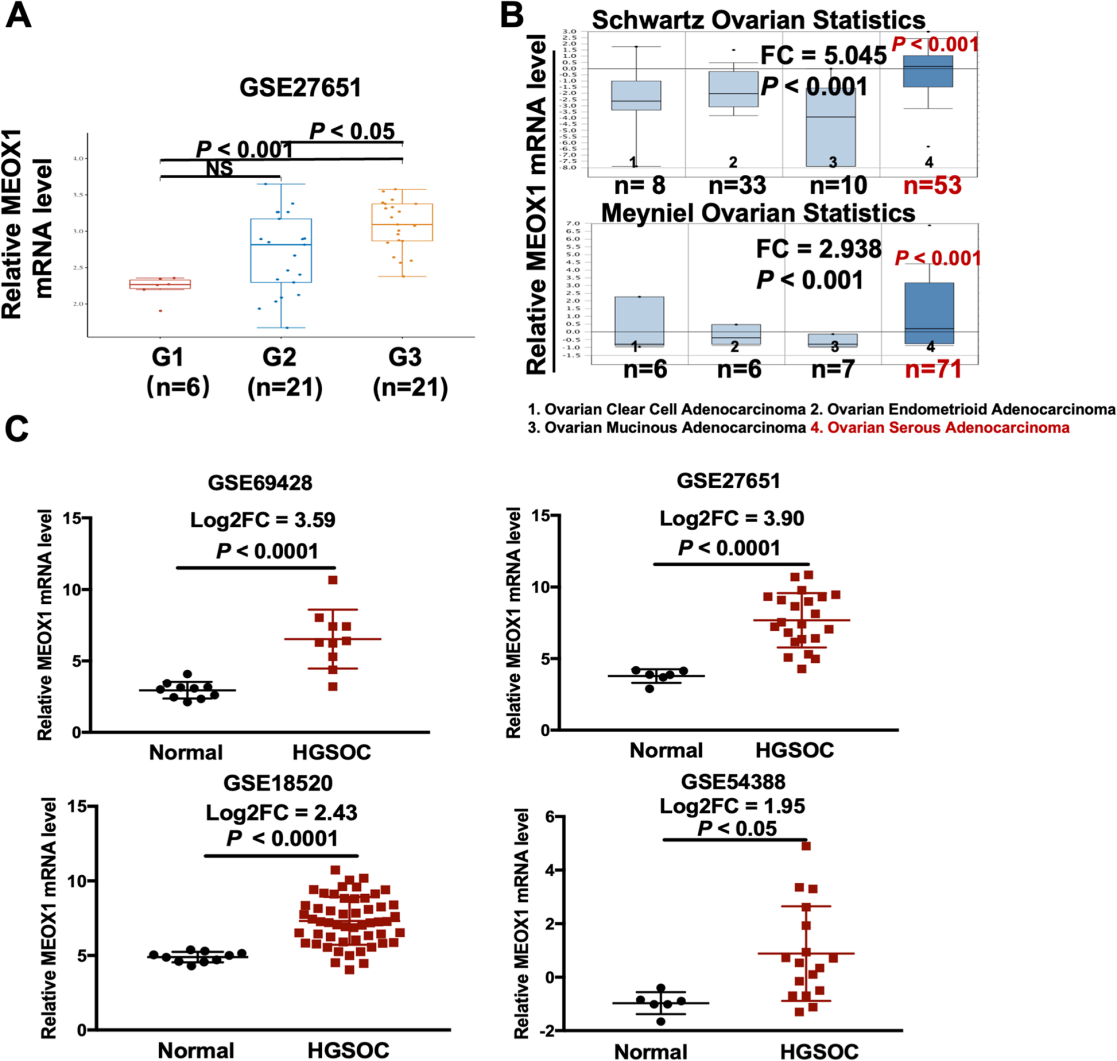
The Expression of MEOX1 in Different Tumor Grade and Various Histopathological Subtypes of Ovarian Cancer. **A** The mRNA expression of MEOX1 in various tumor grade (G stage) of ovarian cancer in GSE27651. NS, not statistically significant. **B** The relative mRNA level of MEOX1 in different histopathological subtypes of ovarian cancer, as determined by the Oncomine database; Schwartz Ovarian Statistics, reporter: U10492_at; Meyniel Ovarian Statistics, reporter: 205619_s_at; *P* < 0.001 vs. Ovarian Mucinous Adenocarcinoma*.* **C** The relative mRNA level of MEOX1 in HGSOC and normal ovarian tissues from GSE69428, GSE18520, GSE54388, and GSE27651

Ovarian cancer is a highly heterogeneous malignant tumor with multiple distinct histopathological subtypes, of which serous ovarian cancer is the most prevalent and has the highest rate of LNM [4, 25]. Analysis of public databases revealed a positive correlation between the mRNA expression of MEOX1 and the tumor grade of ovarian cancer (Figure. 3A). Furthermore, the mRNA level of MEOX1 in serous ovarian adenocarcinoma was significantly higher compared to other histological subtypes (Figure. 3B). In HGSOC datasets (GSE69428, GSE27651, GSE18520, and GSE54388), the mRNA levels of MEOX1 were up to 12, 15, 5, and 3.8 times higher respectively, compared to the corresponding normal ovarian tissues (Figure. 3C). The above findings indicate that higher expression of MEOX1 is associated with increased malignancy in ovarian cancer.

### Bioinformatics analysis showed that MEOX1 was involved in lymph node metastasis of ovarian cancer

**Fig. 4:**
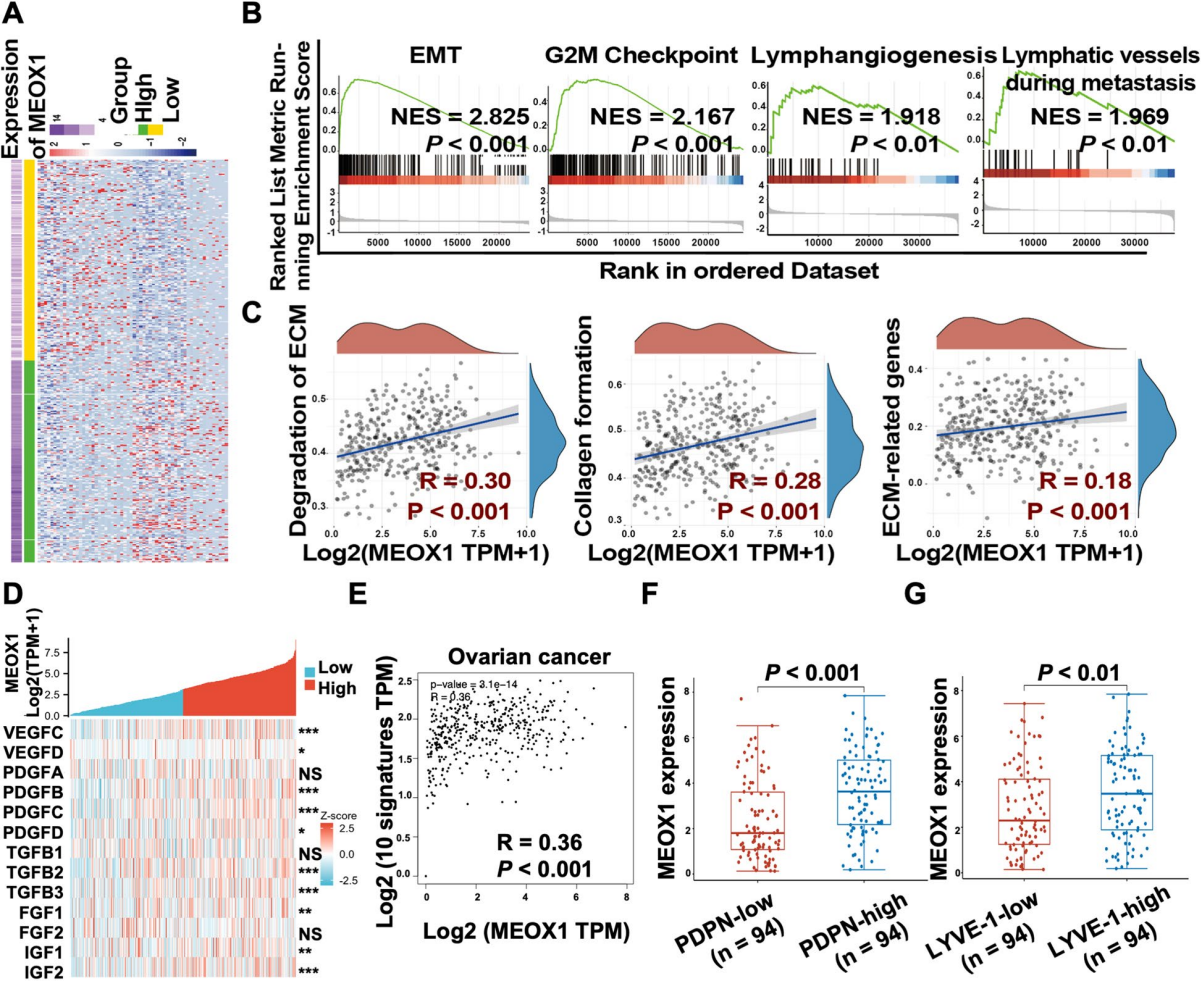
MEOX1 was Associated with LNM of Ovarian Cancer. **A** The heat map of DEGs between TCGA ovarian cancer with higher expression of MEOX1 (MEOX1-high) and ovarian cancer with lower expression of MEOX1 (MEOX1-low). **B** The GSEA analysis of DEGs between MEOX1-high and MEOX1-low TCGA ovarian cancer. **C** Correlation analysis through the Assistant for Clinical Bioinformatics (ACLBI) website between MEOX1 expression and the pathways of "ECM degradation", "collagen formation", and "ECM-related genes" in ovarian cancer. **D** The expression correlation between MEOX1 and prolymphangiogenic factors in TCGA ovarian cancer; NS, not statistically significant, *P < 0.05, **P < 0.01, ***P < 0.001 (Spearman analysis). **E** The correlation analysis via the GEPIA2.0 database between the expression of MEOX1 and the expression of ten prolymphangiogenic factors including VEGF-C, VEGF-D, TGFB2, TGFB3, IGF-1, IGF-2, FGF-1, PDGF-A, PDGF-B, PDGF-C, and PDGF-D in ovarian cancer tissues. **F** The mRNA level of MEOX1 in TCGA ovarian cancer with a lower PDPN expression (PDPN-low) or a higher PDPN expression (PDPN-high). G. The mRNA expression of MEOX1 in TCGA ovarian cancer with a lower LYVE-1 expression (LYVE-1-low) or a higher LYVE-1 expression (LYVE-1-high)

To preliminarily investigate the function and mechanism of MEOX1 in ovarian cancer LNM, we performed gene set enrichment analysis (GSEA) using the expression profiles of TCGA ovarian cancer. The TCGA ovarian cancer cases were divided into two groups based on MEOX1 expression level: MEOX1 high expression and MEOX1 low expression groups, and then the DEGs between these two groups were obtained (Figure. 4A). GSEA results revealed significant enrichment of the obtained DEGs in several processes related to LNM, including epithelial-mesenchymal transition (EMT) (normalized enrichment score (NES) = 2.825), G2M checkpoint (NES = 2.167), lymphangiogenesis (NES = 1.918), and lymphatic vessels during metastasis (NES = 1.969) (Figure. 4B) [11, 12, 26]. Lymphangiogenesis, which is effectively stimulated by prolymphangiogenic factors such as vascular endothelial growth factor (VEGF)-C/D, transforming growth factor-β (TGF-β), insulin-like growth factor (IGF), fibroblast growth factor (FGF), and platelet-derived growth factor (PDGF), is considered a crucial step of LNM [26, 27]. Additionally, the extracellular matrix (ECM) also directly or indirectly induces lymphangiogenesis by providing structural support and nutrient supply for the growth of lymphatic endothelial cells (LECs) [28, 29]. Further analysis showed a positive correlation between MEOX1 expression and ECM composition, as well as the expression of prolymphangiogenic factors. Specifically, MEOX1 expression was positively correlated with the degradation of ECM, collagen formation, and ECM-related genes (Figure. 4C). Moreover, the mRNA expression of MEOX1 was positively correlated with the expression of ten lymphogenic factors, including VEGF-C, VEGF-D, TGFB2, TGFB3, IGF-1, IGF-2, FGF-1, PDGF-A, PDGF-B, PDGF-C, and PDGF-D (Figure. 4D). To further investigate this association, we grouped these ten lymphangiogenic factors into a set of ten signatures and performed expression correlation analysis using the GEPIA2.0 database. As depicted in Figure 4E, a positive linear correlation was observed between MEOX1 expression and the expression of these ten signatures in ovarian cancer, with a correlation coefficient (R) of 0.36. We also examined the association between MEOX1 expression and LECs biomarkers, specifically podoplanin (PDPN) and lymphatic endothelial receptor-1 (LYVE-1) [11].

The results indicated that the mRNA level of MEOX1 was significantly higher in the PDPN high expression group (Figure. 4F) and the LYVE-1 high expression group (Figure. 4G) compared to the PDPN or LYVE-1 low expression group, respectively, suggesting a potential correlation between MEOX1 expression and the abundance of LECs. In summary, it can be hypothesized that MEOX1 may influence lymph node metastasis by regulating tumor growth, tumor EMT, ECM degradation, and lymphangiogenesis.

### MEOX1 was overexpressed in ovarian cancer and was associated with lymph node metastasis

**Fig. 5:**
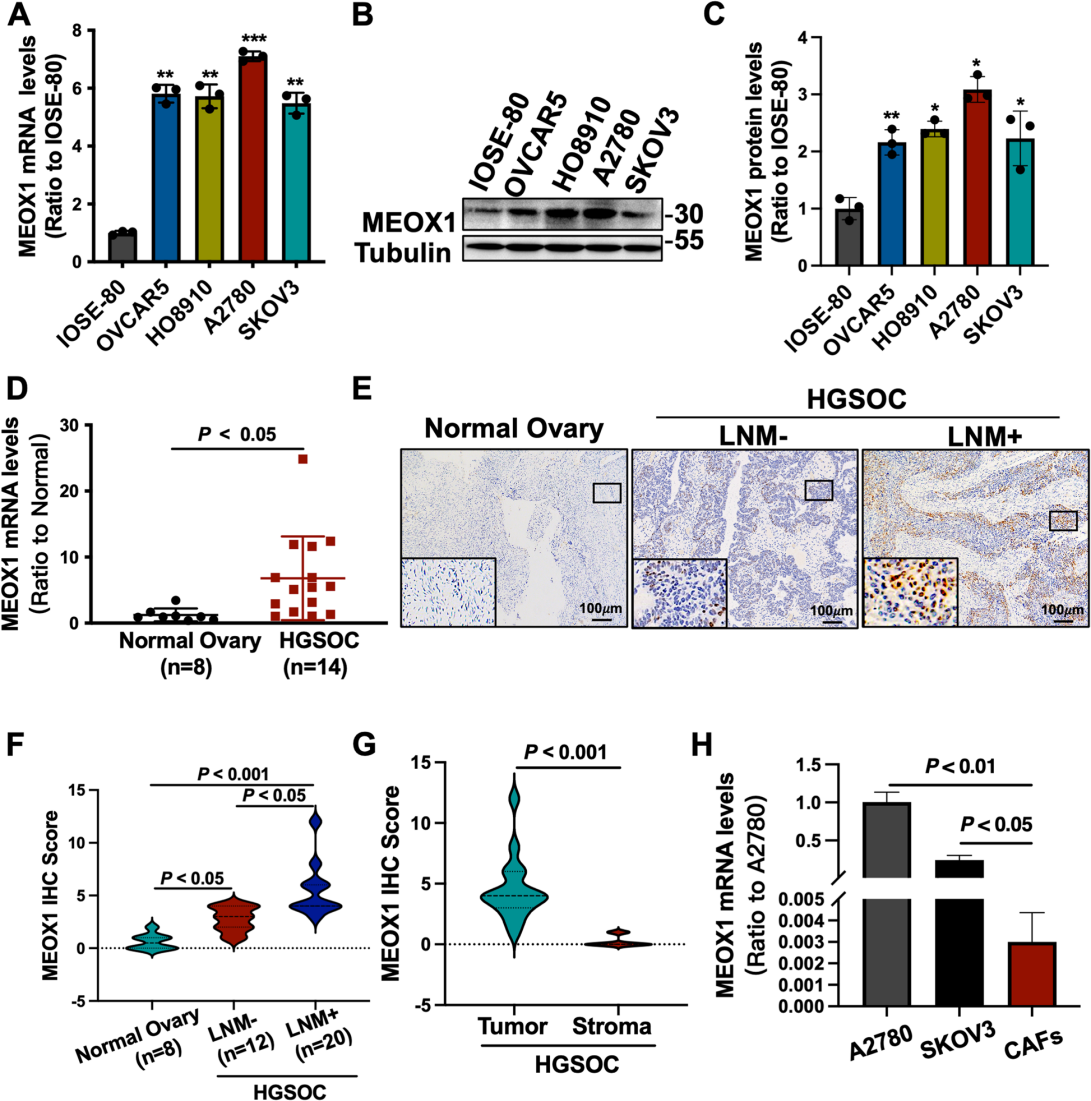
MEOX1 was Abnormally Upregulated in Ovarian Cancer and was Associated with Lymph Node Metastasis. **A**-**C** RT-qPCR assays and western blot assays were used to detect the mRNA levels (**A**) and the protein levels (**B**-**C**) of MEOX1 in normal ovarian epithelial cell line IOSE-80 and ovarian cancer cell lines (OVCAR5, HO8910, A2780, and SKOV3); *P < 0.05, **P < 0.01, ***P < 0.001 vs. IOSE-80. D. RT-qPCR assays were used to detect the relative mRNA expression of MEOX1 in clinical HGSOC samples (*n* = 14) and normal ovarian tissue samples (*n* = 8). **E**–**F** The representative images of MEOX1 IHC staining (E) and statistical results of MEOX1 IHC scores (**F**) in normal ovarian tissue samples (*n* = 8), LNM- HGSOC tissue samples (*n* = 12), and LNM + HGSOC tissue samples (*n* = 20). **G** Statistical results of MEOX1 IHC Score in tumor cells and tumor stroma of 32 HGSOC tissues. **E** The mRNA levels of MEOX1 in A2780, SKOV3, and primary CAFs of ovarian cancer

To demonstrate the overexpression of MEOX1 in ovarian cancer, we initially examined the mRNA and protein expression of MEOX1 in the normal ovarian epithelial cell IOSE-80 and ovarian cancer cell lines (OVCAR5, HO8910, A2780, and SKOV3). Our results showed significantly higher mRNA (Figure. 5A) and protein (Figure. 5B-C) levels of MEOX1 in all ovarian cancer cell lines compared to IOSE-80.

Among the ovarian cancer cell lines, SKOV3 cells had relatively low expression of MEOX1, while A2780 cells had relatively high expression (Figure. 5A-C). Therefore, we chose SKOV3 cells for MEOX1 overexpression and A2780 cells for MEOX1 knockdown treatment. In clinical specimens, we also observed a substantial increase in MEOX1 mRNA expression in primary tumor tissues of HGSOC (*n * = 14) compared to normal ovarian tissues (*n* = 8) (Figure. 5D). Further, we performed immunohistochemistry (IHC) staining of MEOX1 on the collected specimens, including 8 normal ovarian tissue samples, 12 LNM- HGSOC samples, and 20 LNM+ HGSOC samples (Figure. 5E). The IHC analysis revealed stronger staining degree and staining quantity of MEOX1 in tumor tissues of HGSOC, particularly in LNM+ HGSOC, compared to normal ovarian tissues (Figure. 5F). Additionally, MEOX1 protein was primarily localized in the nucleus and predominantly expressed by tumor cells rather than the tumor matrix in ovarian cancer (Figure. 5G). Furthermore, the mRNA level of MEOX1 in ovarian cancer cell lines (A2780 and SKOV3) was remarkedly higher than that in primary ovarian cancer stromal cells cancer-associated fibroblasts (CAFs) (Figure. 5H). Based on these findings, we conclude that MEOX1 is overexpressed in the nucleus of ovarian cancer tumor cells and is associated with LNM in ovarian cancer.

### MEOX1 Promoted the Proliferation of Ovarian Cancer Cells in Vitro and in Vivo

**Fig. 6:**
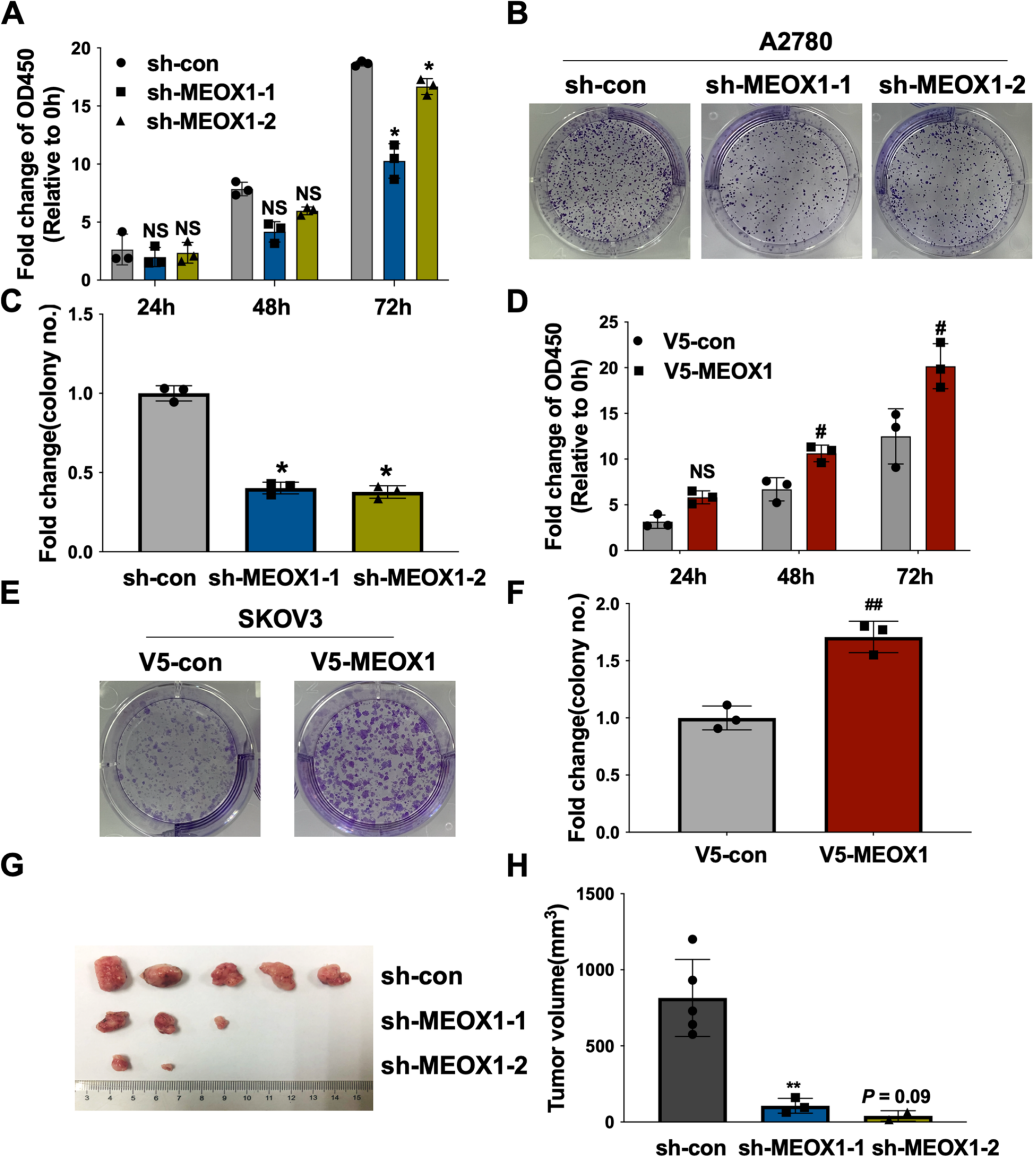
MEOX1 was Involved in the Proliferation of Ovarian Cancer Cells in vitro and in vivo. **A**-**C** The CCK8 assays (**A**) and the colony formation assays (**B**-**C**) revealed the effect of MEOX1 knockdown on the proliferation ability and the colony-forming ability of A2780 cells. **D**-**F** The CCK8 assays (**D**) and the colony formation assays (**E**–**F**) determined the effect of MEOX1 overexpression on the proliferation ability and the colony-forming ability of SKOV3 cells. **G** The images of xenografts harvested at the sacrifice in the sh-MEOX1-1 group, the sh-MEOX1-2 group, and the sh-con group of the nude mice subcutaneous transplant tumor model of ovarian cancer. H. Tumor volume of xenografts in the sh-MEOX1-1 group, the sh-MEOX1-2 group, and the sh-con group, Tumor volume (mm^3^) = length (mm) × width^2^ (mm^2^) /2. NS, not statistically significant, *P < 0.05, **P < 0.01, *P* = 0.09 vs. sh-con; NS, not statistically significant, #*P* < 0.05, ##*P* < 0.01 vs. V5-con

Subsequently, we explored the biological effect of MEOX1 on the proliferation of ovarian cancer cells. Based on the expression level of MEOX1 in A2780 and SKOV3, we selected A2780 and SKOV3 to construct stable MEOX1-overexpressing cell lines (V5-MEOX1) and control cell lines (V5-con), as well as stable MEOX1-suppressing cell lines (sh-MEOX1-1 and sh-MEOX1-2) and control cell lines (sh-con) (Supplementary Figure S5). The knockdown of MEOX1 in A2780 cells resulted in inhibited proliferation at 72 hours (Figure. 6A) and reduced colony-forming ability (Figure. 6B-C) compared to the control group. Conversely, overexpression of MEOX1 in SKOV3 cells significantly increased cell proliferation at 48 and 72 hours (Figure. 6D) and colony-forming capacity (Figure. 6E-F). In the nude mice subcutaneous transplant tumor model using stably MEOX1-suppressing A2780 cells, we observed that knocking down MEOX1 did not affect the survival rate of mice, while compared to the control group (sh-con, 100%), the tumor formation rates of mice in the sh-MEOX1-1 group (60%) and the sh-MEOX1-2 group (40%) were lower (Figure 6G). Furthermore, the tumor volume of xenografts at the endpoint was significantly smaller in the sh-MEOX1-1 group (*P* < 0.01) and the sh-MEOX1-2 group (*P* = 0.09, close to 0.05) than in the control group (Figure. 6H). These findings indicate that MEOX1 promotes the proliferation of ovarian cancer cells both *in vivo* and *in vitro*.

### MEOX1 Promoted EMT of Ovarian Cancer Cells in Vitro

**Fig. 7:**
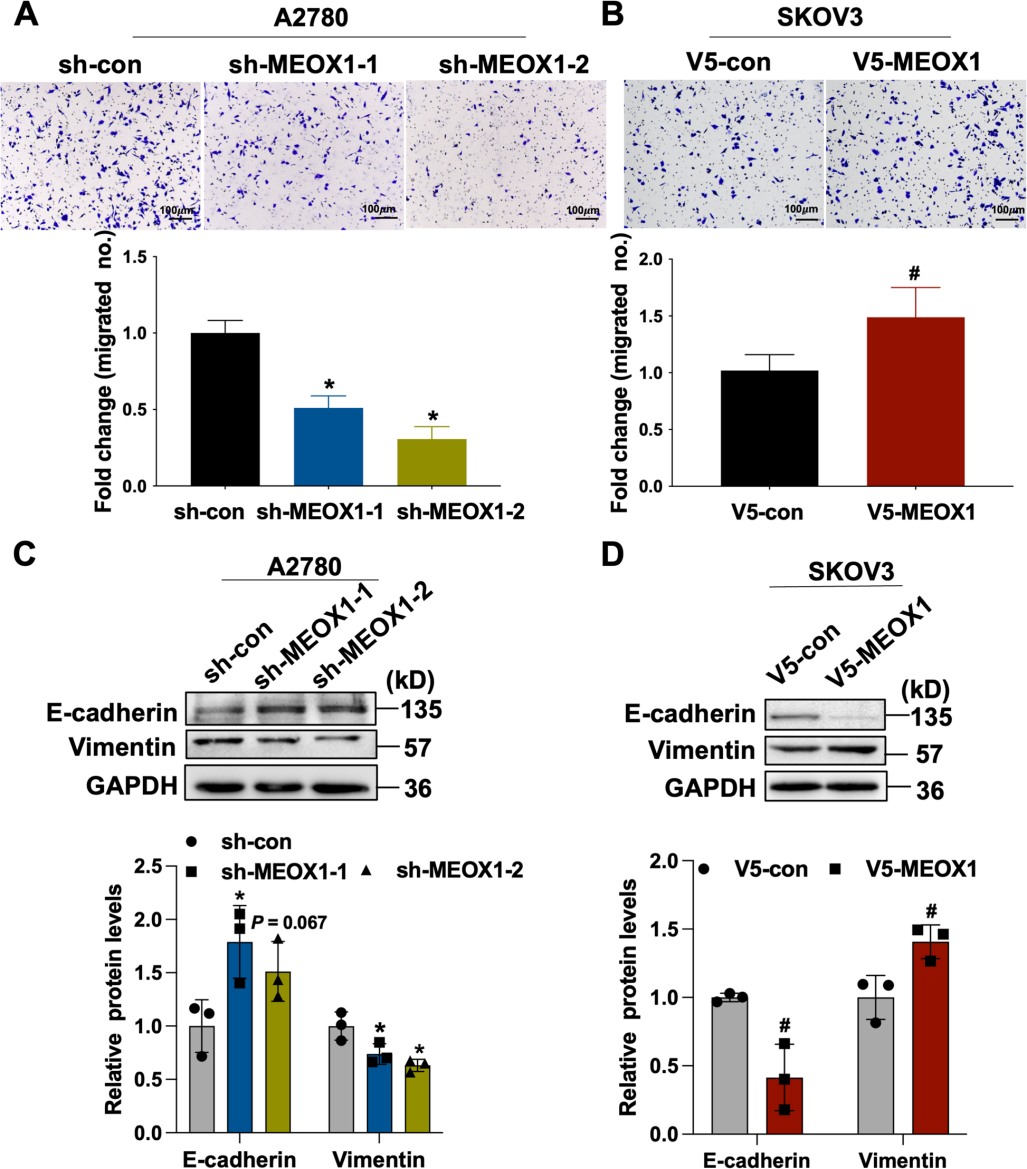
MEOX1 regulated EMT of Ovarian Cancer Cells in vitro. **A**-**B** Transwell migration assays were conducted to evaluate the migratory potential of A2780 cells (**A**) treated with or without MEOX1 suppression and SKOV3 cells (**B**) treated with or without MEOX1 overexpression. The upper image displayed the migrated cells, and the lower image showed the statistical results of the migrated cell number. **C**-**D** Western blot assays were performed to determine the effect of MEOX1 knockdown (**C**) or overexpression (**D**) on the expression of E-cadherin and Vimentin proteins in A2780 cells or SKOV3 cells; the lower image showed the statistical results of the protein expression of E-cadherin and Vimentin; *P < 0.05, *P* = 0.067 vs. sh-con; #*P* < 0.05 vs. V5-con

Since the “EMT pathway” had the highest enrichment score based on the GSEA analysis of MEOX1 in ovarian cancer, we continued to determine the *in vitro* effect of MEOX1 on the EMT of ovarian cancer cells. Transwell migration assays demonstrated that suppressing MEOX1 significantly impaired the migratory potential of A2780 cells (Figure. 7A), while overexpressing MEOX1 significantly enhanced the migration of SKOV3 cells (Figure. 7B). Additionally, western blot assays revealed that knocking down MEOX1 increased the expression of E-cadherin and decreased the expression of Vimentin in A2780 cells (Figure. 7C); In contrast, overexpression of MEOX1 led to a decrease in E-cadherin expression and an increase in Vimentin of SKOV3 cells (Figure. 7D). These findings provide further evidence that MEOX1 regulates EMT in ovarian cancer cells at the cellular level.

## Discussion

LNM is a prevalent occurrence in ovarian cancer and has a significant impact on clinical staging and patient prognosis [2–4]. Based on the principle of individualized and precision treatment, it is crucial to identify specific and reliable biomarkers for LNM in ovarian cancer, which will not only help determine the presence of LNM but also offer novel treatment strategies for ovarian cancer. Utilizing publicly accessible multi-platforms for genomic data mining can be highly advantageous in the research of ovarian cancer LNM due to its high efficacy and minimal cost [13, 14]. Previous studies have shown that the majority of genetic abnormalities found in lymphatic metastases were already present in the primary tumor, with isolated mutations or variants being rare in metastatic lesions [23, 24]. These suggest that LNM-related genes may predominantly exist in the primary tumor. Therefore, we conducted data mining using expression profile data of ovarian cancer primary lesions from public databases to identify new LNM-related genes of ovarian cancer. Our findings revealed MEOX1 as a novel LNM-related gene in ovarian cancer, which can predict the occurrence of LNM and poor clinical prognosis in ovarian cancer patients.

Dermatofibrosarcoma protuberans is an aggressive spindle cell neoplasm that is known for its expression of MEOX1 [22]. In breast cancer, MEOX1 is highly expressed in tumor tissues and is associated with poor overall survival, advanced tumor stage, and lymph node metastasis; Functional experiments in breast cancer cells have confirmed that MEOX1 triggers trastuzumab resistance, EMT, and self-renewal of breast cancer stem cells [19]. Recent research has identified MEOX1 as a potential therapeutic target for suppressing the growth of p53- and PTEN-deficient triple-negative breast cancer through the JAK/STAT signaling pathway [30]. Moreover, MEOX1 has been found to be upregulated in non-small cell lung cancer (NSCLC) and is positively associated with lymph node metastasis, advanced clinical staging, and unfavorable survival in NSCLC patients; In vitro studies have also shown that upregulation of MEOX1 enhances cell proliferation and colony-forming abilities [18]. Nonetheless, a more recent investigation has demonstrated that MEOX1 is downregulated in lung cancer and serves as an anti-carcinogenic gene [21]. In ovarian cancer, MEOX1 has been reported to function as a cofactor of pre-B-cell leukemia homeobox-1 (PBX1), mediating the cancer-promoting biological behavior of PBX1 [20]. The preceding research indicates that MEOX1 may be a new target for the occurrence and progression of tumor, but its function in cancer is heterogeneous.

Through pan-cancer analysis, we discovered that MEOX1 expression was generally lower in tumor tissues of most cancer types compared to corresponding normal tissues, such as BRCA and LUSC, which was not fully aligned with previous reports [18, 19]. Remarkably, we observed that MEOX1 was overexpressed in tumor tissues of ovarian cancer, and the expression of MEOX1 in ovarian cancer tissues and cell lines ranked first among all cancer types, suggesting that MEOX1 may play a greater role in the occurrence and development of ovarian cancer. Among various histopathological subtypes, serous ovarian cancer is the most prevalent subtype, with HGSOC not only comprising the greatest proportion but also exhibiting the highest degree of malignancy and being more prone to LNM [4, 25]. It was reported that the incidence rate of LNM in HGSOC patients was the highest (67.1%), with the incidence rate in patients with low-grade serous ovarian cancer (LGSOC) ranking second (58.8%) [4]. Bioinformatic analyses revealed that serous ovarian adenocarcinoma had the highest level of MEOX1 expression compared to other histopathological subtypes. Furthermore, the mRNA expression of MEOX1 increased with higher tumor grade in ovarian cancer. Based on the above observations, we hypothesize that the elevated expression of MEOX1 is associated with the malignant behavior of ovarian cancer.

Since MEOX1 was initially identified through a search of public databases, it is necessary to verify its expression and association with LNM using clinical samples and conduct functional experiments to enhance the validity of the conclusion. Consistent with the results of bioinformatics analysis, we demonstrated that the mRNA and protein levels of MEOX1 were higher in clinical ovarian cancer specimens and ovarian cancer cell lines than in normal ovarian tissues and the normal ovarian epithelial cell line IOSE-80, respectively. Moreover, we found that MEOX1, as a transcription factor, was primarily localized in the nucleus of cancer cells, with minimal expression in the tumor matrix. Notably, the expression level of MEOX1 was substantially higher in LNM+ ovarian cancer tissues compared to LNM- ovarian cancer tissues, providing additional evidence that the expression of MEOX1 had a certain predictive value in determining the presence of LNM. The evidence presented above suggested that the anomalous overexpression of MEOX1 may reflect the lymph node metastasis potential of ovarian cancer cells and could be a crucial molecular event in the progression of the disease.

LNM involves a complex series of dynamic interactions between tumors and the host, including possesses such as lymphangiogenesis, tumor detachment from the primary tumor, tumor spread and metastasis, and ECM remodeling [11, 12]. Growing evidence suggests that lymphangiogenesis, primarily induced by multiple prolymphangiogenic factors like VEGF-C, VEGF-D, and PDGF, is essential for initiating LNM [26, 27]. Neonatal lymphatic vessels are an extremely early predictor of the occurrence of LNM in tumors, playing an indispensable role in the initial dissemination of malignant tumors [11, 12]. Clinical observations have shown that the greater the density of lymphatic vessels in ovarian cancer tissues, the higher the risk of peritoneal metastasis, distant metastasis, and LNM [31]. Additionally, ECM degradation promotes lymph node metastasis of tumor cells by disrupting adhesions between tumor cells and adjacent cells, releasing ECM-isolated growth factors [32]. As anticipated, our bioinformatics analysis revealed that MEOX1 was not only associated with malignant behaviors of ovarian cancer such as tumor proliferation and EMT, but also closely linked to lymphangiogenesis, lymphatic vessels during metastasis, and degradation of ECM. Besides, the expression of MEOX1 in ovarian cancer tissues was positively correlated with the expression of multiple prolymphangiogenic factors, such as VEGF-C, PDGF, FGF, IGF, etc. Our fundamental experiments also substantiate the effect of MEOX1 on tumor growth and EMT of ovarian cancer. The above suggests that MEOX1 may participate in the LNM process of ovarian cancer by affecting various biological behaviors, such as tumor growth, tumor EMT, ECM degradation, and lymphangiogenesis. However, further functional experiments are required to determine the specific function of MEOX1 in promoting LNM in ovarian cancer and whether MEOX1, as a transcription factor, promotes lymphangiogenesis by regulating the expression of prolymphangiogenic factors of ovarian cancer cells.

In conclusion, we identified MEOX1 as a novel LNM-related biomarker in ovarian cancer by mining the TCGA and GEO databases. We found that upregulation of MEOX1 in ovarian cancer was associated with LNM, high G stage, and an unfavorable prognosis; functional experiments showed that MEOX1 promoted the proliferation and EMT of ovarian cancer cells. Furthermore, bioinformatics analyses hinted at the possibility that MEOX1 might be implicated in the LNM of ovarian cancer by regulating tumor proliferation, tumor EMT, lymphangiogenesis, and ECM remodeling. However, the underlying molecular mechanism is still elusive and requires additional research. Overall, our preliminary findings may provide a valuable diagnostic marker and potential therapeutic target for managing LNM in ovarian cancer.

## Materials and methods

### Microarray data acquisition

The primary data of ovarian cancer RNA-sequencing profiles and corresponding clinical information were acquired from the TCGA database (http://portal.gdc.cancer.gov/). A total of 146 patients were obtained according to the following criteria: comprehensive gene expression data; exclusive clinical data, including LNM status, age, race, histological type, clinical stage, and vital status. 99 patients were LyI+, while 47 were LyI-.

Three gene expression profiling datasets, including mRNA expression data of HGSOC samples and matched normal ovarian tissues, were obtained from the GEO database (https://www.ncbi.nlm.nih.gov/geo/). The number of HGSOC samples vs. normal tissues in the GSE18520, GSE54388, and GSE27651 datasets was 53 vs. 10, 16 vs. 6, and 22 vs. 6, respectively.

### Data mining for LNM-related DEGs and HGSOC-related DEGs

The LNMS-related DEGs (LyI+ vs. LyI-) were screened using the edgeR package, and the threshold for a significant difference was set to |log2 Fold Change | > 0.585 (that is, Fold Change > 1.5 or Fold Change < 1/1.5) and *P*-value < 0.05. Then, DEGs mining of the four GEO profiles (HGSOC vs. normal) was performed under the same conditions using the limma package. DEGs co-existing in all three GEO profiles with the same down- or upregulation trend were defined as the final cancer-related DEGs of ovarian cancer. By overlapping the upregulated LNM-related DEGs and the upregulated cancer-related DEGs, a total of 12 overlapping genes were obtained.

### Validation of the expression of the overlapping 12 genes and its relationship with prognosis in ovarian cancer

The gene expression levels of the overlapping 12 genes in ovarian cancer tissues and normal ovarian tissues were verified using data from the TCGA and GTEx databases (https://gtexportal.org/). A fold change > 2 and a *P*-value < 0.05 were considered significant differences. The ROC analysis was performed to assess the predictive ability of the overlapping 12 genes in predicting the sample state (normal or tumor) and lymph node metastatic state (LNM+ and LNM-). The value of AUC is between 0.5 and 1. The closer the AUC is to 1, the better the diagnostic effect. AUC has low accuracy when it is 0.5-0.7, AUC has certain accuracy when it is 0.7-0.9, and AUC has high accuracy when it is above 0.9.

The survival analysis was conducted via Kaplan–Meier plotter (http://kmplot.com/analysis/) to achieve the OS and PFS analysis of the expression of the overlapping 12 genes in ovarian cancer. A *P*-value < 0.05 was regarded as statistically significant.

### Gene expression of MEOX1 and its association with prognosis in pan-cancer via the public databases

The pan-cancer expression landscape of MEOX1 mRNA was obtained via the UALCAN (http://ualcan.path.uab.edu/analysis-prot.html) and TIMER2.0 (http://timer.cistrome.org/) website. Via the GEPIA2.0 (http://gepia.cancer-pku.cn), we analyzed the differences in mRNA expression of MEOX1 between tumor tissues (TCGA data) and normal tissues (GTEx data). Using the Oncomine (http://oncomine.org/) platform, we examined the mRNA expression of MEOX1 in various tumor tissues (Su Muti-cancer statistics, reporter: 36010_at) and various tumor cell lines (Staunton Cellline Statistics, reporter: U10492_at). The CCLE database (https://sites.broadinstitute.org/ccle) rendered the mRNA expression of MEOX1 in pan-cancer cell lines, including ovarian cancer. The relation between MEOX1 expression and clinical survival in pan-cancer was obtained from the “Pan-Cancer” module on the ACLBI (https://www.aclbi.com/) website.

### The association of MEOX1 expression with tumor grade and histopathology of ovarian cancer

The mRNA level of MEOX1 in different histopathological types of ovarian cancer was obtained from the Oncomine (Schwartz Ovarian Statistics, reporter: U10492_at; Meyniel Ovarian Statistics, reporter: 205619_s_at). The relationship between MEOX1 expression and G stage in ovarian cancer in GSE27651 was obtained from the GEO database.

### GSEA and pathway analysis of MEOX1 in ovarian cancer

According to the average expression level of MEOX1, the ovarian cancer cases in TCGA were categorized into a MEOX1 high expression group (MEOX1-high, *n* = 107) and a MEOX1 low expression group (MEOX1-low, n = 272). Then, enrichment of DEGs between MEOX1-high and MEOX1-low was performed by the GSEA analysis using the GSEA software and Hallmark signatures EMT, G2M checkpoint, “PID_Lymph_Angiogenesis_Pathway”, and “CLASPER_Lymphatic_Vessels_During_Metastasis_Up”. Enriched datasets with a false discovery rate (FDR) < 0.05 and *P*-value = 0.01 were considered statistically significant.

The expression correlation between MEOX1 and pathways in ovarian cancer was analyzed via the ACLBI website: after obtaining the expression profile data of ovarian cancer in the TCGA database, download all gene sets included in the relevant pathways such as EMT markers, degradation of ECM, ECM-related genes, and collagen formation; single sample GSEA algorithm was used to calculate the enrichment score of the samples in the relevant pathways, and the correlation between gene expression and enrichment score was calculated to obtain the correlation between the gene and the pathway.

### The expression correlation of MEOX1 with prolymphangiogenic factors or lymphatic endothelial cell markers in ovarian cancer

Downloaded and normalized the transcriptome sequencing data of ovarian cancer from the TCGA database, used Spearman correlation analysis method to analyze the expression correlation between MEOX1 and prolymphangiogenic factors, and finally used ggplot package (https://www.xiantao.love/) to visualize the correlation heat maps. Then, logged in to GEPIA2.0 online database website, selected "Exploration Analysis" - "Correlation Analysis" module, input MEOX1 and the ten signatures of prolymphangiogenic factors, selected Spearman analysis method and chose TCGA ovarian cancer, and the correlation between them could be obtained. Based on the expression levels of lymphatic endothelial cell markers, TCGA ovarian cancer was sorted into the high-expression group and the low-expression group. Then analyze whether there was a significant difference in mRNA levels of MEOX1 between the two groups.

### Cell lines and cell culture

The human ovarian cancer cell lines SKOV3, A2780, and OVCAR-5 were obtained from the American Type Culture Collection (ATCC, Virginia, USA), and HO8910 cells were purchased from the Cell Bank of the Chinese Academy of Sciences (Shanghai, China). CAFs were separated and cultured from the ovarian cancer tissues obtained from the Obstetrics & Gynecology Hospital of Fudan University with written informed consent from patients. The acquirement of all clinical samples used in our study was approved by the institute's Ethics Committee of the Obstetrics & Gynecology Hospital of Fudan University. SKOV3 and A2780 cells were maintained in RPMI 1640 (SH30809.01, HyClone, Logan, Utah, USA) containing 10% fetal bovine serum (FBS) (10270-106, GIBCO, California, USA) and 1 × 10^5^ IU/L penicillin and streptomycin (15140-122, GIBCO, California, USA). HO8910 and OVCAR-5 cells were cultured in DMEM (SH30022.01, HyClone, Logan, Utah, USA) supplemented with 10% FBS and 1 × 10^5^ IU/L penicillin and streptomycin. CAFs were cultured in Fibroblast Medium (2301, ScienCell, California, USA). All cells were incubated at 37 °C in a 5% CO2 and 95% air humidified atmosphere.

### Suppression or overexpression of MEOX1 in ovarian cancer cells

To downregulate the expression of MEOX1 in ovarian cancer cells, we used Lentivirus (LV)-MEOX1-shRNA1/2 (MEOX1-sh1/2) (Genechem, Shanghai, China) to transfect the cells according to the manufacturer’s protocol. The target sequence of MEOX1-shRNA-1 was GGATGAAGTGGAAGCGTGTGA; the target sequence of MEOX1-shRNA-2 was GGAGGAGCACATCTTCACTGA. Conversely, ovarian cancer cells were transfected with LV-MEOX1(Genechem, Shanghai, China) to overexpress MEOX1.

### Quantitative Real-Time PCR (qPCR)

According to the manufacturer's protocol, total RNA was isolated using Total RNA Extraction Reagent (R401-01, Vazyme, Nanjing, China). Reverse transcription was performed using a ReverTra Ace qPCR RT Master Mix (FSQ101, TOYOBO, Osaka, Japan). qPCR was performed with SYBR Green Realtime PCR Master Mix (QPK-201, TOYOBO, Osaka, Japan). The primers used in these studies were: MEOX1 (forward: 5’ -GCAGGGGGTTCCAAGGAAA- 3’, reverse: 5’-GTCAGGTAGTTATGATGGGCAAA-3’) and GAPDH (forward: 5’- GGAGCGAGATCCCTCCAAAAT -3’, reverse: 5’-GGCTGTTGTCATACTTCTCATGG -3’).

### Western blot assays

Proteins were extracted from cells using RIPA buffer supplemented with phenylmethylsulfonyl fluoride and a protease inhibitor cocktail. The BCA protein assay kit (P0012, Beyotime Biotechnology, Shanghai, China) was used to determine the protein concentration. Protein samples were separated using SDS-PAGE. Then separated proteins were electrophoretically transferred onto PVDF membranes. After blocking with 5% non-fat milk for 1 h at room temperature, we incubated the membranes with a primary antibody overnight at 4 °C and then incubated them with a secondary antibody for 1 h at room temperature. Chemiluminescence on the protein bands was revealed using High-sig ECL Western Blotting Substrate (180-5001, Tanon Science & Technology Ltd, Tanon, Shanghai, China). The primary antibodies used in the present study were anti-MEOX1 (TA804716, Origene, Wuxi, China), anti-Tubulin (11224-1-AP, Proteintech, Chicago, USA), anti-Vimentin (#5741, Cell Signaling Technology, Danvers, MA, USA), anti-E-cadherin (#3195, Cell Signaling Technology, Danvers, MA, USA), and anti-GAPDH (#5174, Cell Signaling Technology, Danvers, MA, USA).

### Immunohistochemistry (IHC)

All clinical samples used in our study were obtained with written informed consent from patients. The institute's Ethics Committee of the Obstetrics & Gynecology Hospital of Fudan University approved the present study (protocol code 2021-94, May 6th, 2021). After heating for 1 h at 65 °C, the paraffin-embedded slides were deparaffinized in xylene and rehydrated in graded ethanol solutions (100%-70%). Then, the slides were subjected to 1% Triton X-100 and 3% hydrogen peroxide at room temperature for 10 minutes in sequence, followed by antigen retrieval using the Antigen Retrieval Buffer (50 × Tris-EDTA, pH 9.0) (36318ES60, Yeasen, Shanghai, China) at 95 °C according to the instructions. Slides were blocked with 5% donkey serum for 1 h at room temperature and then washed three times with PBST (PBS with 0.05% Tween 20). For detection of MEOX1, slides were incubated with a primary antibody against MEOX1 (ab279366, Abcam, Cambridge, UK) overnight at 4 °C. After being washed three times with PBST, the slides were incubated with a secondary antibody at room temperature for 1 h. Next, the slides were overlaid with DAB Horseradish Peroxidase Color Development Kit (G1212-200T, Servivebio, Wuhan, China) according to the instructions after three washes with PBST. Finally, slides were counterstained with hematoxylin, dehydrated, and mounted. For each slide, at least three randomly selected microscopic fields were chosen for quantitative analysis of IHC staining. The evaluation of MEOX1 expression was based on the staining score including the percentage of positive cells in the tissue (0, 0%; 1, 1–10%; 2, 11–50%; 3, 51–70%; 4, 71–100%) and the staining intensity (0, none; 1, weak; 2, moderate; 3, strong).

### Transwell migration assay

The migration of ovarian cancer cells was evaluated using the 8 μm pore size, 24-well polycarbonate membrane (353097, Becton,Dickinson and Company, New Jersey, USA). Cells (5 × 10^4^ cells/well) in a conditioned medium were seeded in the upper chamber with the lower chamber supplemented with the double-serum medium. After 24 h, non-migrating cells on the surface of the upper chamber were removed with a cotton swab. Migrating cells on the lower surface were fixed with 4% paraformaldehyde (BL539A, Biosharp, Guangzhou, China), stained with 0.5% Crystal Violet Stain Solution (60506ES60, Yeasen, Shanghai, China), and counted in five random fields per well under an optical microscope.

### Cell counting kit-8 (CCK8) assay

Cell proliferation was evaluated using the Cell Counting Kit-8 (C0039, Beyotime Biotechnology, Shanghai, China). A2780 or SKOV3 cells were seeded in 96-well plates after down- or upregulating MEOX1 expression. After culturing for a particular time (24 h, 48 h, 72 h), CCK-8 was added into the medium and incubated for 1 h at 37 °C. The proliferation was measured by a microplate reader (BIO-TEK, Vermont, USA).

### Colony formation assay

Cells were plated in six-well plates (2 × 10^3^ per well), and the medium was refreshed every three days. Ten days later, the colonies were visible and then fixed in 4% paraformaldehyde (BL539A, Biosharp, Guangzhou, China) and stained with 0.5% Crystal Violet Stain Solution (60506ES60, Yeasen, Shanghai, China). Finally, the stained colonies (> 10 cells) were counted using a light microscope.

### Tumor xenograft

All animal experiments complied with ethical regulations and were approved by the Animal Welfare and Ethics Group, Department of Experimental Animal Science, Fudan University (Code Number: 202209013S). After being stably transfected with LV-MEOX1-shRNA1/2 or LV-control-shRNA, A2780 cells (5 × 10^6^ in 100 μl PBS) were subcutaneously injected into the 5-6 weeks nude mice (GemPharmatech, Nanjing, China). Approximately one week later, tumors were detectable, and tumor volumes were measured and calculated by the formula V = (maximal diameter × perpendicular diameter^2^) / 2. After 4 weeks, the mice were euthanized, the transplanted tumor was taken and measured.

### Statistical analysis

All values were presented as mean ± S.D and were analyzed and plotted using GraphPad Prism 9.0. The Student's t-test was used to determine whether there was a significant difference between the two groups; One way ANOVA was used to determine whether there was a significant difference between multiple groups. *P* < 0.05 was considered statistically significant.

### Supplementary Information

**Supplementary Material 1.**

**Supplementary Material 2.**

**Supplementary Material 3**

**Supplementary Material 4.**

**Supplementary Material 5.** 

**Supplementary Material 6.**

**Supplementary Material 7.**

## Data Availability

No datasets were generated or analysed during the current study.

## Abbreviations

LNM: Lymph node metastasis
HGSOC: High-grade serous ovarian cancer
MEOX1: Mesenchyme homeobox 1
TCGA: The Cancer Genome Atlas
GEO: Gene Expression Omnibus
BRCA1: Breast cancer susceptibility protein 1
LNM +: LNM positive
LNM-: LNM negative
FIGO: Federation of International of Gynecologists and Obstetricians
DEGs: Differentially expressed genes
OS: Overall survival
PFS: Progression-free survival
ROC: Receiver operating characteristic
AUC: Area under the curve
CI: Confidence interval
UALCAN: University of ALabama at Birmingham Cancer Data Analysis Portal
CCLE: Cancer Cell Line Encyclopedia
TIMER2.0: Tumor Immune Estimation Resource Version 2
BLCA: Bladder urothelial carcinoma
BRCA: Breast invasive carcinoma
CESC: Cervical squamous cell carcinoma and endocervical adenocarcinoma
COAD: Colon adenocarcinoma
KICH: Kidney chromophobe
KIRP: Kidney renal papillary cell carcinoma
LUAD: Lung adenocarcinoma
LUSC: Lung squamous cell carcinoma
READ: Rectum adenocarcinoma
STAD: Stomach adenocarcinoma
THCA: Thyroid carcinoma
UCEC: Uterine corpus endometrial carcinoma
CHOL: Cholangiocarcinoma
KIRC: Kidney renal clear cell carcinoma
LIHC: Liver hepatocellular carcinoma
OV: Ovarian cancer
DLBC: Diffuse large B-cell lymphoma
LAML: ACute myeloid leukemia
LGG: Brain lower-grade glioma
SARC: Sarcoma
TGCT: Testicular germ cell tumors
THYM: thymoma
UCS: Uterine carcinosarcoma
SKCM: Skin cutaneous melanoma
GEPIA2.0: Gene Expression Profiling Interactive Analysis version 2
VEGF-C: Vascular endothelial growth factor C
VEGF-D: Vascular endothelial growth factor D
TGF-β: Transforming growth factor β
IGF-1: Insulin-like growth factor 1
IGF-2: Insulin-like growth factor 2
FGF-1: Fibroblast growth factor 1
FGF-2: Fibroblast growth factor 2
PDGF-B: Platelet derived growth factor B
PDGF-C: Platelet derived growth factor C
PRAD: Prostate adenocarcinoma
IHC: Immunohistochemistry
HPA: Human Protein Atlas
GSEA: Gene Set Enrichment Analysis
NES: Normalized enrichment score
EMT: Epithelial-mesenchymal transition
ACLBI: Assistant for Clinical Bioinformatics
ECM: Extracellular matrix
HLECs: Human lymphatic endothelial cells
NSCLC: Non-small cell lung cancer
PBX1: Pre-B-cell leukemia homeobox-1
CAFs: Cancer-associated fibroblasts
TAMs: Tumor-associated macrophages
ATCC: American Type Culture Collection
FBS: Fetal bovine serum
qPCR: Quantitative Real-Time PCR
CCK8: Cell counting kit-8
FDR: False discovery rate
