# Supplementary figures and images for: Unraveling the molecular mechanisms of lymph node metastasis in ovarian cancer: focus on MEOX1
Source: J Ovarian Res. 2024 Mar 14;17:61. doi: 10.1186/s13048-024-01384-6 (PMC10938838; doi:10.1186/s13048-024-01384-6)

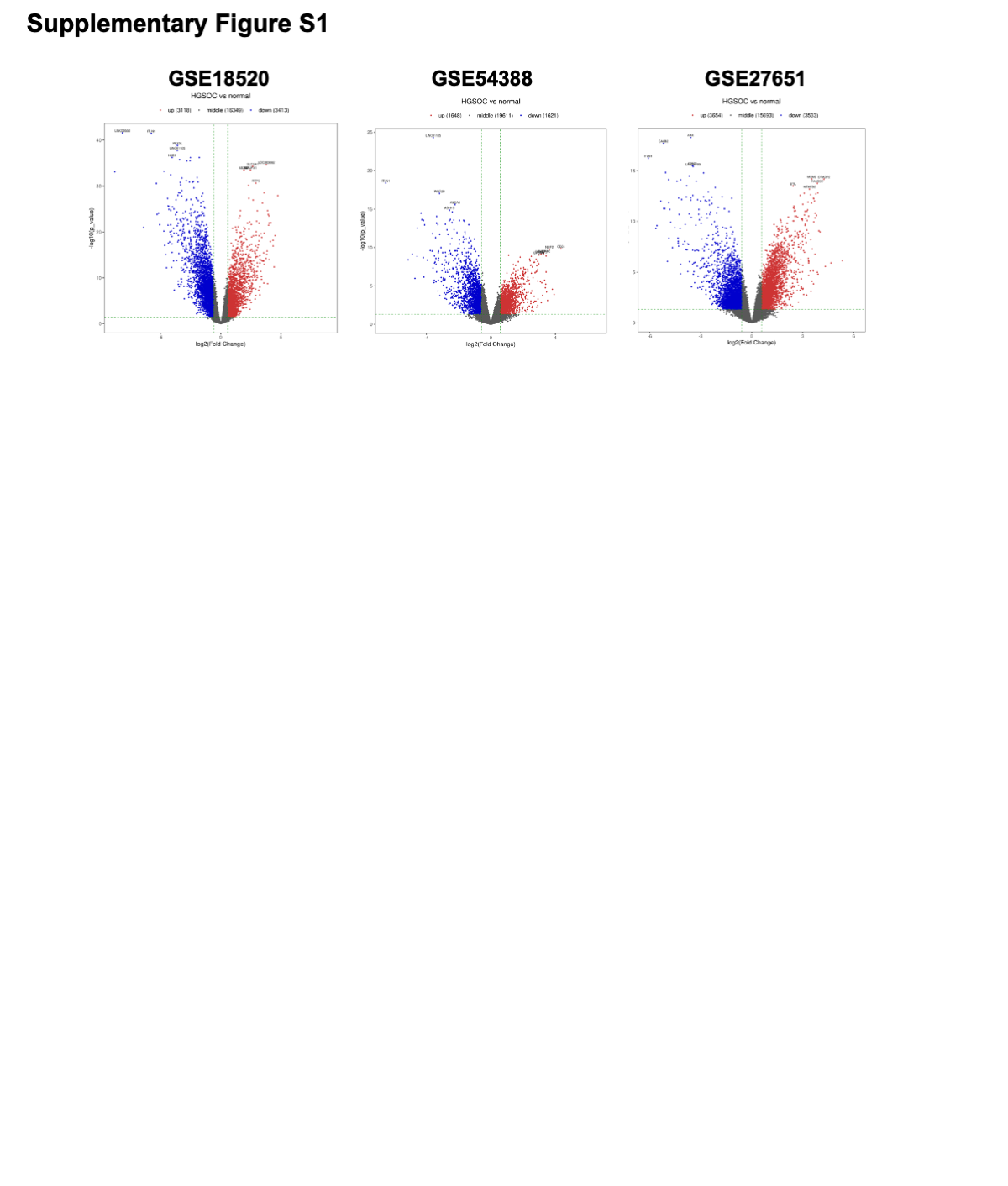

Supplement: Supplementary file 3 — Supplementary Material 3 [file 13048_2024_1384_MOESM3_ESM.tiff]

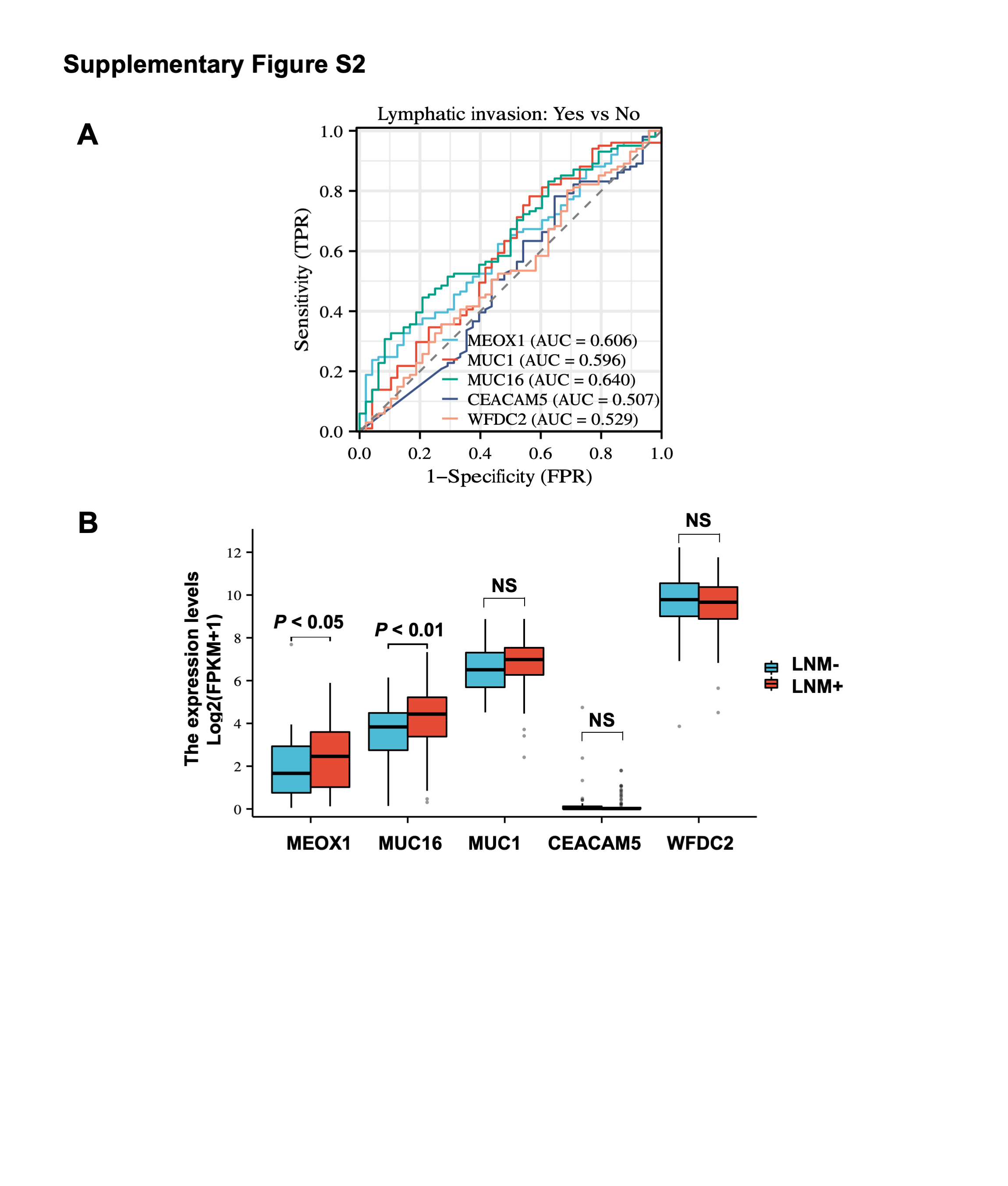

Supplement: Supplementary file 4 — Supplementary Material 4. [file 13048_2024_1384_MOESM4_ESM.tiff]

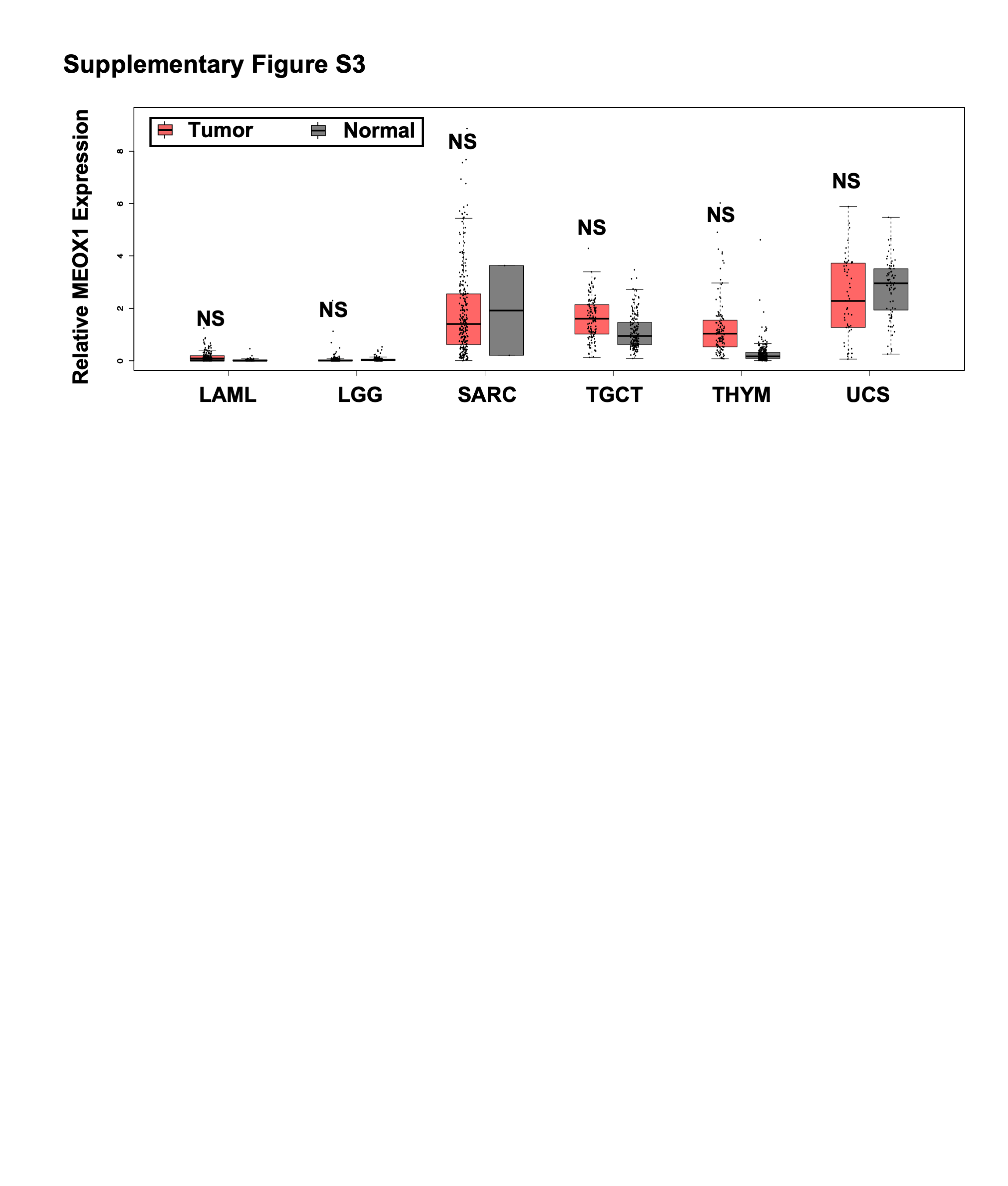

Supplement: Supplementary file 5 — Supplementary Material 5. [file 13048_2024_1384_MOESM5_ESM.tiff]

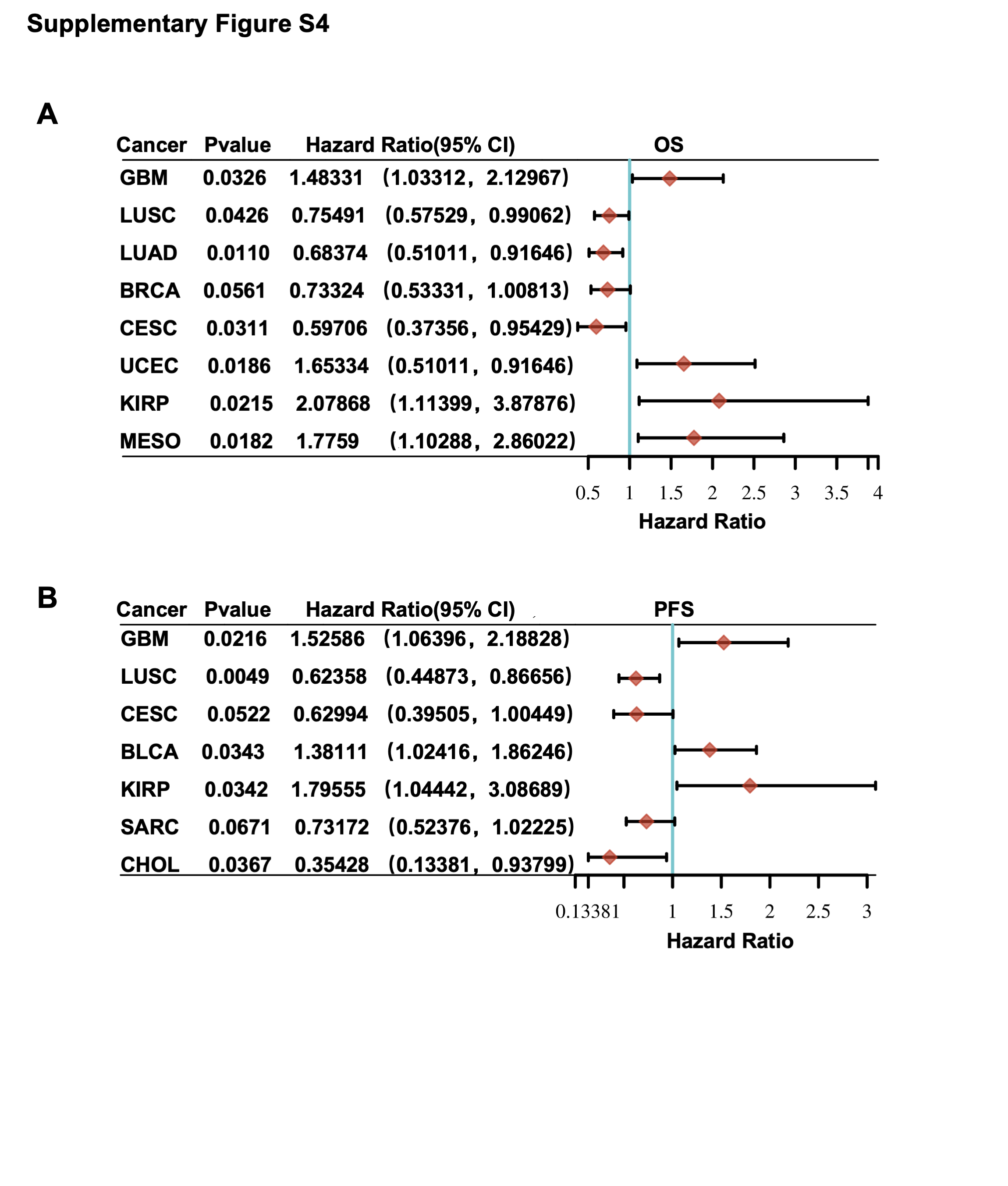

Supplement: Supplementary file 6 — Supplementary Material 6. [file 13048_2024_1384_MOESM6_ESM.tiff]

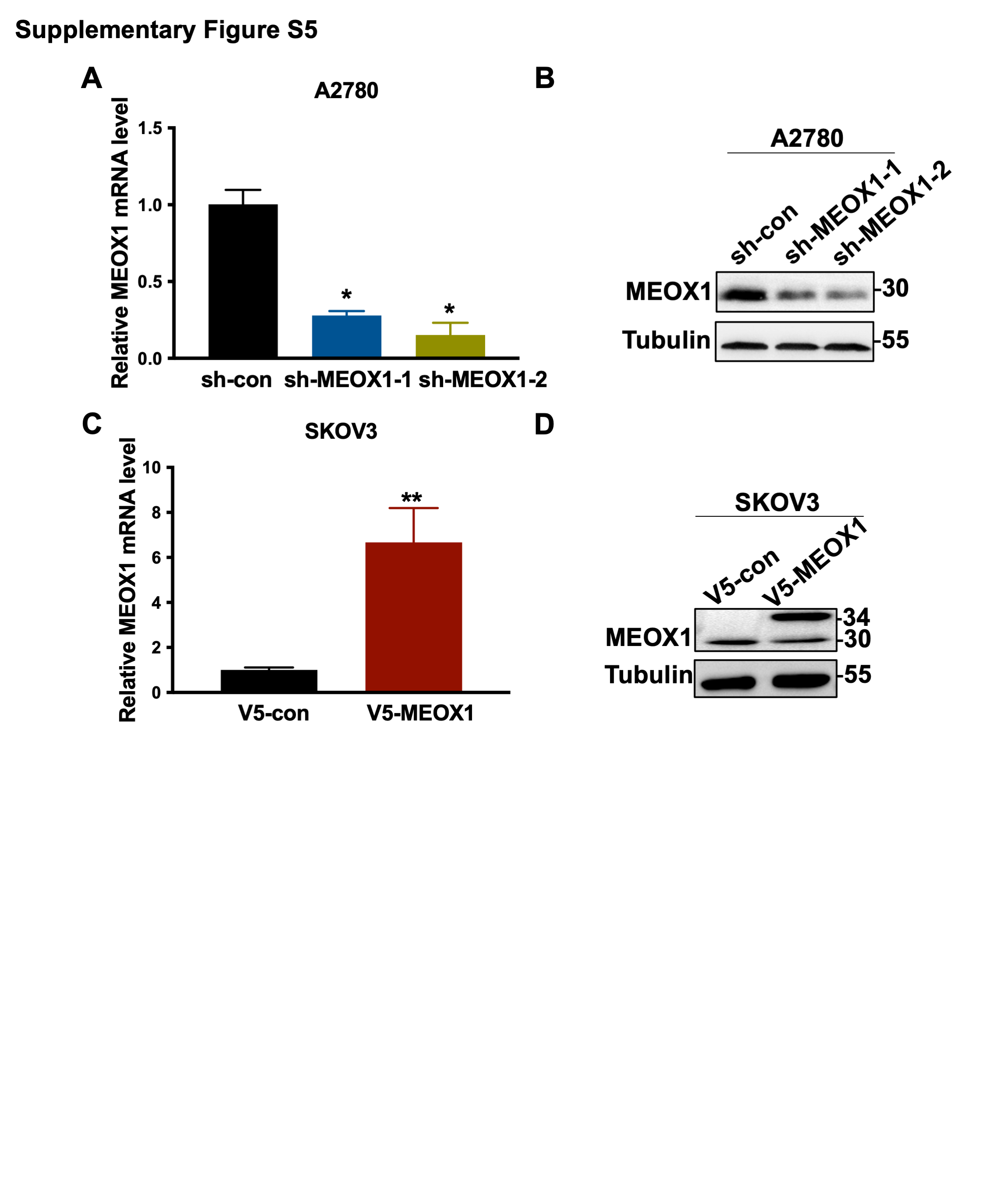

Supplement: Supplementary file 7 — Supplementary Material 7. [file 13048_2024_1384_MOESM7_ESM.tiff]
